# Antipsychotic drug use during pregnancy and neonatal outcomes: a systematic review and meta-analysis

**DOI:** 10.1007/s00737-025-01651-5

**Published:** 2026-01-08

**Authors:** Dervla Quinn, Michael Donnelly, Ciaran O’Neill

**Affiliations:** Centre for Public Health (CPH), Queen’s University Belfast, Belfast, United Kingdom

**Keywords:** Antipsychotics, Neonatal outcomes, Systematic review, Meta-analysis

## Abstract

**Purpose:**

The use of antipsychotics during pregnancy has increased over the past two decades, primarily driven by an increase in the use of second-generation antipsychotic drugs. However, knowledge regarding the reproductive safety of antipsychotic drugs remains limited. This systematic review and meta-analysis investigated the associations between in utero antipsychotic drug exposure and congenital malformations and other neonatal outcomes.

**Methods:**

A systematic search of MEDLINE, Embase, and PsycInfo was conducted from database inception to February 2024 for cohort and case-control (English language) studies that examined maternal antipsychotic exposure and reported risk estimates for one or more of the following outcomes: congenital malformation, preterm birth, low birth weight, stillbirth, or neonatal intensive care unit admission. Study quality was assessed using the Newcastle-Ottawa Scale, and reporting was guided by the PRISMA statement and MOOSE guidelines. Pooled estimates were calculated using a random-effects model.

**Results:**

Twelve studies (comprising over 10 million pregnancies across 12 countries) met the inclusion criteria. A pooled meta-analysis of eight studies indicated borderline evidence of an association between the risk of congenital malformations and in utero antipsychotic drug exposure, with moderate heterogeneity (odds ratio [OR] 1.27; 95% confidence interval [CI] 0.996–1.624, *p* = 0.0535; I^2^ = 53%). No association was observed when limited to second-generation antipsychotics (OR 1.16; 95% CI 0.78–1.72, *p* = 0.47). Regarding the outcome of preterm birth, antipsychotic exposure was associated with an increase in risk (OR 1.35; 95% CI 1.13–1.62, *p* < 0.01), though there was moderate to high heterogeneity (I^2^ = 70%). There was insufficient data to perform a meta-analysis for the other outcomes.

**Conclusion:**

Meta-analyses did not indicate strong evidence that in utero antipsychotic exposure is a major teratogen; and although an association was observed between maternal antipsychotic use and preterm birth, there was significant heterogeneity across studies. The decision to continue antipsychotic use during pregnancy involves a complex balancing of risks and benefits for women and their healthcare professionals. Any potential risks to the developing foetus must be weighed against the risks of discontinuing treatment, including the possibility of relapse in women with severe mental illness, which can have serious consequences for a woman and her infant. Finally, there is a need for further robustly designed studies.

**Supplementary Information:**

The online version contains supplementary material available at 10.1007/s00737-025-01651-5.

## Article highlights

Only higher-quality studies that adjusted for other potential risk factors were included.

Antipsychotic use in pregnancy was associated with a small increase in the risk of preterm birth.

There was no strong evidence that antipsychotics act as major teratogens.

Further large studies are needed to better understand the potential risks associated with individual antipsychotics.

**Supplementary Information:**

The online version contains supplementary material available at 10.1007/s00737-025-01651-5.

## Introduction

Over the past two decades, the prevalence of antipsychotic use during pregnancy has increased in many countries, largely driven by an increase in second-generation antipsychotic use (Toh et al., 2013; Park et al. 2017; Reutfors et al. 2020). Despite this trend, knowledge regarding the reproductive safety of antipsychotics remains limited. There is evidence that antipsychotics can cross the placenta (Newport et al. 2007), and safety warnings related to an increased risk of withdrawal symptoms and extrapyramidal signs in neonates exposed to antipsychotics in the third trimester have been previously issued (Medicines and Healthcare products Regulatory Agency 2011). Given the lack of available evidence from randomised controlled trials, observational studies are considered the most pragmatic approach to address gaps in knowledge (Abel 2013).

Previous systematic reviews and meta-analyses have assessed potential risks associated with in utero antipsychotic exposure. Coughlin et al. (2015) conducted a systematic review and meta-analysis examining a range of obstetric and neonatal outcomes, and identified associations between antipsychotic exposure and several adverse neonatal outcomes including major congenital malformations, preterm delivery, small for gestational age, and low birth weight. However, they noted that the analysis was limited by the lack of adjustment for potential confounding factors in the included studies. More recently, Joseph-Delaffon (2024) also reported an association between in utero antipsychotic exposure and several neonatal outcomes, including preterm birth. Wang, Brauer, et al. (2021) conducted a systematic review and meta-analysis focusing on the risk of congenital malformations following prenatal antipsychotic exposure and reported a pooled adjusted risk ratio (aRR) of 1.23 (95% confidence interval (CI): 0.96–1.58, *p* = 0.173). Since these reviews, several large cohort studies have been published, adding to the body of evidence on the risks of antipsychotic drug use during pregnancy. The aim of this systematic review and meta-analysis is to update and summarise the available evidence on the association between antipsychotic use during pregnancy and the risk of congenital malformations, as well as other neonatal outcomes, including preterm birth, low birth weight (LBW), stillbirth, and admission to the neonatal intensive care unit (NICU).

## Materials and methods

### Search strategy

A comprehensive literature search was conducted using MEDLINE (Ovid), Embase (Ovid), and PsycInfo (American Psychological Association; Ovid) from database inception through 21 February 2024 (detailed search strategy in supplementary information). A library specialist provided input on the search strategy.

The reporting of this study was guided by the standards of the Preferred Reporting Items for Systematic reviews and Meta-Analyses (PRISMA 2020) statement (Page et al. 2021) and the Meta-analysis of Observational Studies in Epidemiology (MOOSE) guidelines (Stroup et al. 2000). The study protocol was registered with PROSPERO, an international prospective register of systematic reviews (CRD42024536272).

### Study selection

Cohort and case-control studies published in the English language that examined any type of maternal antipsychotic use, including first-generation antipsychotics (FGA) and second-generation antipsychotics (SGA) were included. Studies that included a comparator group of unexposed women and provided sufficient information to estimate an effect size (with standard error) were eligible for inclusion. Outcomes of interest included congenital malformations, preterm birth (< 37 weeks of gestation), low birth weight (LBW, defined as birth weight less than 2,500 g), stillbirth, and NICU admission. Meeting abstracts, letters, editorials, commentaries, case reports, and case series were excluded. Reference lists of relevant studies were also screened for potential inclusions. A sample of approximately 10% of titles and abstracts was screened independently by two reviewers, with a third reviewer adjudicating on conflicts. Concordance exceeded 80% in the sample and the remainder of the screening was completed by the first reviewer. This process was repeated for full-text review. Study screening and calculation of inter-rater reliability were conducted using Covidence (‘Covidence systematic review software’, 2024).

### Quality assessment and data extraction

Data extraction was conducted using a standardised form, and included year of publication, study location, study period, data source, study design, sample size, selection of study group, pregnancy gestation definition, medication exposure definition, antipsychotic class (FGA, SGA, or both), exposure period, selection of comparison group, outcome definition and ascertainment, potential confounding factors, statistical analysis and risk estimates with 95% confidence intervals.

The quality of studies was assessed using the Newcastle-Ottawa Scale for case-control and cohort studies (Wells et al.,). Quality assessment covered three domains: selection of the study group, comparability of the groups, and ascertainment of outcomes for cohort studies or ascertainment of exposure for case-control studies. For a study to score a point in the comparability domain, effect estimates had to be adjusted, consistent with criteria used by Wang, Brauer, et al. (2021). Studies with a total score of at least 6, including at least one point in each domain, were included in the meta-analysis. Quality assessment and data extraction were performed by one reviewer, with a second reviewer reviewing a sample of approximately 10% of studies. Disagreements were resolved by consensus.

### Data analysis

Adjusted risk estimates and corresponding confidence intervals were extracted and log-transformed to calculate standard errors. Relative risks (RR) and odds ratios (OR) were treated as equivalent measures of risk, given the relatively low prevalence of the outcomes. A random-effects model was pre-specified to produce pooled estimates due to anticipated between-study heterogeneity. Estimates were pooled using the DerSimonian–Laird random-effects model. Heterogeneity between studies was assessed using the Cochran Q test, and the I^2^ statistic to measure the degree of inconsistency across studies. Meta-analyses were performed when more than three risk estimates were available. For the outcome of congenital malformations, subgroup analyses included by antipsychotic class (FGA and SGA), individual antipsychotics, and geographic location (by continent). Two post hoc sensitivity analyses were conducted; one excluding studies that included chromosomal abnormalities in the outcome of congenital malformations or that did not restrict antipsychotic exposure during pregnancy to the first trimester; and a second using a risk estimate from a study included in a previously published meta-analysis. A funnel plot was used to assess potential publication bias, and an analysis was conducted by sequentially omitting each study to evaluate its effect on pooled estimates. All meta-analyses were performed using Stata statistical software, version 18 (StataCorp 2023).

## Results

**Fig. 1:**
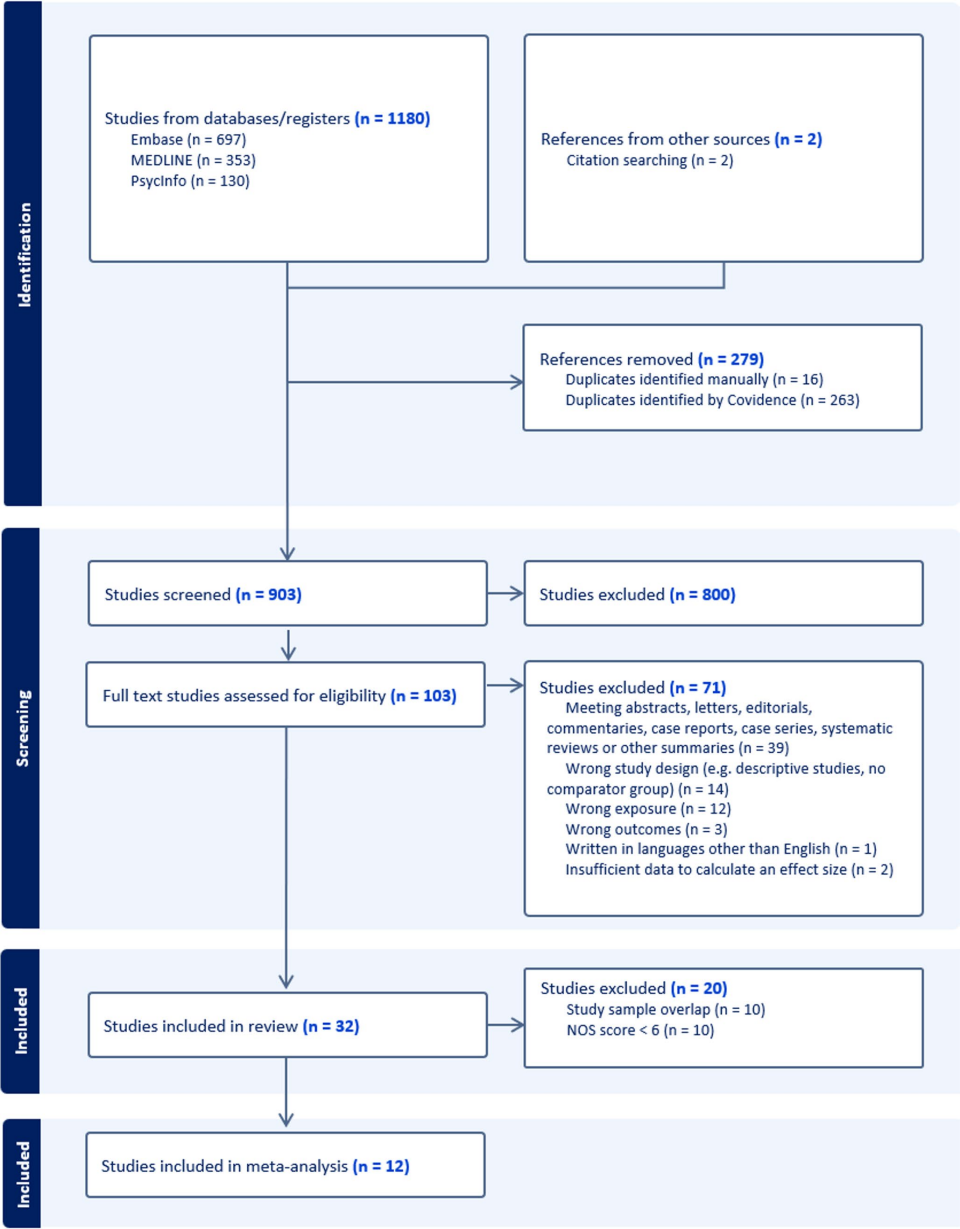
PRISMA flow diagram

After duplicate removal, 903 titles were screened. There was a high level of agreement between the first reviewer (D.Q.) and the second reviewer (C.O’N.) during the title and abstract screening, with a proportion agreement of 94.4% and a Cohen’s Kappa of 0.88 in a sample of records. Discrepancies in seven titles were resolved by a third reviewer (M.D.). A total of 103 records underwent full-text eligibility assessment, with complete agreement between the first and second reviewers in a sample of records (100% proportion agreement). The PRISMA flow diagram is presented in Fig. 1.

Thirty-two studies were included in the systematic review, study characteristics are presented in Table 1. Of these, twelve studies were eligible for inclusion in the meta-analysis.

**Table 1:** Characteristics of studies included in systematic review

Reference	Year published	Study location	Study period	Data source	Study design	Sample size	Selection of study group	Pregnancy gestation definition
Slone et al.	1977	USA	Not reported	Collaborative Perinatal Project	Cohort	Overall: 50,282 pregnant women Exposed: 1,309	Not described.	Not reported
Rumeau-Rouquette et al.	1977	France	1963 - 1969	Prospective survey.	Cohort	Overall: 12,764 Exposed: 315	Pregnant women attending for maternity care in 12 hospitals in Paris.	Not reported
Diav-Citrin et al.	2005	Israel, Germany, The Netherlands, Italy	1989 - 2001	Four teratogen centres	Cohort	Exposed: 215 Comparison: 631	Women contacting a teratology information service	Not reported
McKenna et al.	2005	Israel, Canada, UK	Not reported	Two teratogen centres; prescription event monitoring.	Cohort	Exposed: 151	**Canada/Israel**: Women contacting a teratology information service. **UK**: Drug Safety Research Unit database.	Not reported
Reis et al.	2008	Sweden	1995 - 2005	Swedish national registers	Cohort	Overall: 958,729 Exposed: 2,830	All births in Swedish Medical Birth Register	Gestation mostly derived from 2^nd^ trimester ultrasound
Lin et al.	2010	Taiwan	2001-2003	Taiwan National Health Insurance Research Database (NHIRD)	Cohort	-696 women with schizophrenia (236 FGA users; 46 SGA) -matched with 3,480 women without chronic illness	Women who had singleton live births between 2001 - 2003	Not reported
Boden et al.	2012	Sweden	2005 - 2009	Three Swedish national databases	Cohort study	Olanzapine & clozapine: 169 Other antipsychotics: 338 No antipsychotics: 357,696	All women giving birth 2005 - 2009	Primarily based on ultrasound, or recorded LMP.
Habermann et al.	2013	Germany	1997 - 2009	Teratology Information Service (questionnaires at 1^st^ contact & 8 weeks after birth).	Cohort	SGA: 561 FGA: 284 (“cohort I”) Unexposed: 1,122 (“cohort II”)	Women contacting a teratology information service	Weeks after LMP.
Sadowski et al.	2013	Canada	2005 - 2009	Teratology information service (Motherisk Program)	Cohort	SGA: 133 (from 370 initial contacts) Matched unexposed: 133	Women contacting a teratology information service	Not reported.
Bellet et al.	2015	France	2004 - 2011	Teratogen information services (Terappel & Paris TIS).	Cohort	Aripiprazole: 86 in exposed group (from 323 initial contacts) Matched unexposed: 172	Women contacting a teratology information service. Known teratogens excluded.	Not reported.
Sørensen et al.	2015	Denmark	1997 - 2008	National registries	Cohort	Overall: 1,005,319 pregnancies Antipsychotics: 3.164	All pregnancies (excluding induced abortion in exposed)	From date of conception (calculated from gestation age at outcome).
Sutter-Dallay et al.	2015	France	2001 - 2010	13 Psychiatric Mother Baby Units (MBU database)	Cohort	Overall: 1,071 Exposed: 267	Women admitted to MBUs with infants <1 year	Not reported
Vigod et al.	2015	Canada	2003 - 2012	Linked population databases	Matched cohort study	Overall: >50,000 1,021 in each matched group	Singleton deliveries	Conception date calculated from gestational age at birth
Cohen et al.	2016	USA	2008 - 2014	National Pregnancy Registry for Atypical Antipsychotics (3 maternal interviews, records)	Cohort	Enrolled: 487 Exposed: 214 Unexposed: 89	Women aged 18-45 with a history of psychiatric disorders who agreed to participate.	Not reported
Huybrechts et al.	2016	USA	2000 - 2010	Medicaid database	Cohort	Overall: 1,341,715 Antipsychotics: 9,258	Pregnancies with a live-born infant 1^st^ trimester known teratogen exposed excluded	Not reported
Petersen et al.	2016	UK	1995 - 2012	CPRD & THIN primary care electronic health databases	Cohort	Overall: 319,520 Group A: 416 Group B: 670 Group C: 318,434	Pregnancies identified from GP records and infants linked with household identifier (65-75%).	Start of pregnancy LMP or 280 days before delivery if no records suggested a different duration of pregnancy.
Frayne et al.	2017	Australia	2007 - 2013	Women with serious mental illness attending a maternity hospital	Cohort	Overall: 268 No psychotropic medications: 67 Antipsychotics: 87 Antidepressants: 55 Both: 59	Singleton pregnancies. Excluded other psychiatric medications.	Not reported.
Cohen et al.	2018	USA	2008 - 2017	National Pregnancy Registry for Atypical Antipsychotics (3 maternal interviews, records)	Cohort	Enrolled: 888 Exposed: 152 Unexposed: 205	Women aged 18-45 with a history of psychiatric disorders who agreed to participate.	Not reported
Anderson et al.	2020	USA	1997 - 2011	National Birth Defects Prevention Study (interviews after delivery)	Case control	Cases: 31,651 Controls: 11,615	Cases: major structural birth defects with unknown aetiologies	Pregnancy based on estimated date of conception.
Ellfolk et al.	2020	Finland	1996 - 2016	National registers	Cohort	Overall: 1,181,090 SGA: 4,225 FGA: 1,576 Unexposed: 21,125	Singleton pregnancies.	Start of pregnancy based on gestational age at birth (primarily from ultrasound). Trimesters: up to 84 days, 85-182, 183 to birth).
Ellfolk et al.	2021	Finland	1996 - 2017	National registers	Cohort	Overall: 1,273,987 SGA: 3,478 FGA: 1,030 Unexposed: 22, 540	Singleton pregnancies ending in livebirth, stillbirth or elective termination due to malformation (exposures to known teratogens excluded; n=9,876).	Start of pregnancy based on gestational age at birth (primarily from ultrasound). First trimester: LMP to 84 days
Freeman et al.	2021	USA	2008 - 2020	National Pregnancy Registry for Atypical Antipsychotics (3 maternal interviews, records)	Cohort	Enrolled: 1,906 Exposed: 158 Unexposed: 690	Women aged 18-45 with a history of psychiatric disorders who agreed to participate.	Not reported
Viguera et al.	2021	USA	2008 - 2020	National Pregnancy Registry for Atypical Antipsychotics (3 maternal interviews, records)	Cohort	Enrolled: 1,906 Exposed: 621 Unexposed: 690	Women aged 18-45 with a history of psychiatric disorders who agreed to participate.	Not reported
Wang et al.	2021	Hong Kong	2001 - 2019	CDARS	Cohort	411,251 mother-child pairs	All live births between 2001 - 2015 in mothers aged 15 -50 (antidepressant and lithium exposed pregnancies excluded)	Gestational age at birth based on ultrasound. LMP estimated by subtracting gestational age from date of delivery. 1^st^ trimester: 0 – 90 days after LMP 2^nd^ trimester: 91 – 180 after LMP 3^rd^ trimester: 181 days after LMP to delivery
Heinonen et al.	2022	Sweden	2006 - 2017	Swedish national registers	Cohort	Overall: 1,307,487 Exposed: 2,677	All singleton births (pre-pregnancy diabetes & valproate excluded)	Not reported
Lin et al.	2022	Taiwan	2010 - 2016	Taiwanese Health & Welfare Data	Cohort	Overall: 800,866 Exposed: 1,619 Unexposed: 1,619	Women aged 18-49 years with a first pregnancy resulting in a live singleton birth (pre-existing diabetes and no outpatient visits excluded)	Not reported
Yakuwa et al.	2022	Japan	2005 - 2016	Pregnant women who contacted teratology information service (questionnaire one month after birth)	Cohort	Overall: 4,250 Exposed: 351 Comparison: 3,899	Singleton live births of women who contacted Japan Drug Information Institute & completed a questionnaire 1 month after birth	LMP estimated from reported date of pregnancy outcome and gestational age.
Cohen et al.	2023	USA	2008 – 2021	National Pregnancy Registry for Atypical Antipsychotics (3 maternal interviews, records)	Cohort	Enrolled: 2,293 Lurasidone: 134 Quetiapine: 264 Unexposed: 886	Women aged 18-45 with a history of psychiatric disorders who agreed to participate.	Not reported
Huybrechts et al.	2023	5 Nordic countries & USA	1996 - 2018	Health registers (InPress Consortium)	Cohort	Overall: 6,483,446 Unexposed: 6,455,324 SGA: 21,751 FGA: 6,371	*Nordic:* All pregnancies resulting in singleton liveborn infants *USA*: Medicaid data of mothers linked to live born infants (exposure to known teratogen excluded)	Not reported
Kananen et al.	2023	Finland	2002 - 2016	Hospital register study (KuBiCo)	Cohort	Overall: 36,083 Quetiapine: 152 Any antipsychotic: 227 Unexposed: 35,133	All singleton births (≥22 weeks & weight≥500g) in Kuopio Hospital	Not reported
Liu et al.	2023	Denmark	2008 - 2017	Danish national registers	Cohort	Overall: 503,158 Antipsychotics: 1,252	All singleton pregnancies viable at week 11	Start of pregnancy estimated from gestational age from ultrasound. LMP used if no ultrasound.
Viguera et al.	2023	USA	2008 - 2022	National Pregnancy Registry for Psychiatric Medications (3 maternal interviews, records)	Cohort	Enrolled: 2,619 Exposed: 49 Unexposed: 1,156	Women aged 18-45 with a history of psychiatric disorders who agreed to participate.	Not reported

Reference	Medication exposure	FGA, SGA or both	Exposure period	Selection of comparison group	Outcome ascertainment & definition	Potential confounding factors	Statistical analysis
Slone et al.	Phenothiazines	FGA	First 4 lunar months of pregnancy (congenital malformations).	Not exposed to phenothiazines.	Ascertainment not described. Grouped into uniform (includes major) and nonuniform.	Results are reported as standardised, but methods not further described.	Not described in detail. *Subgroups*: heavy vs. other exposure
Rumeau-Rouquette et al.	Phenothiazines	FGA	3 months after LMP.	Women unexposed to phenothiazines in first trimester.	Blinded assessment of infants by paediatricians. Chromosomal and hereditary malformations excluded.	None	One sided tests (not further described).
Diav-Citrin et al.	Haloperidol & penfluridol	FGA	Any time during pregnancy (78.2% in 1^st^ trimester).	Women counselled during pregnancy for exposures considered to be non-teratogenic	Follow-up by telephone +/- questionnaire with woman/doctor/midwife (between neonatal up to 6 years). Premature birth: ≤37 weeks	Considered maternal age and smoking for preterm birth (final estimates do not appear to be adjusted).	**Main analysis**: Χ2/Fisher exact test. Mann-Whitney test. **Post hoc**: linear & logistic regression to analyse the relative contribution of predictors.
McKenna et al.	SGA (olanzapine, risperidone, quetiapine, clozapine)	SGA	All exposed in first trimester.	**Canada/Israel:** Women counselled during pregnancy for exposures considered to be non-teratogenic and without psychiatric history (Canada only). Matched to cases by maternal age (+/- 2 years) and gestational age (+/- 2 weeks). **UK:** matched to “similar” women in DSRU database.	Follow-up by telephone with woman +/- questionnaire with doctor. Preterm < 37 weeks.	No adjustment.	**Main analysis**: Χ2/Fisher exact test. Student t test/Mann-Whitney U test.
Reis et al.	Any antipsychotic in ATC N05A recorded at first antenatal appointment (lithium reported separately)	Both	Early pregnancy (first anti natal appointment)	All women who gave birth.	Congenital malformations from all 3 sources. Preterm < 37 weeks LBW < 2,500g (from paediatric record)	Year of birth, maternal age, parity, smoking, previous miscarriages	**Main analysis**: Mantel Haenszel method. Exact Fisher tests if low numbers. Two exposure groups: (1) Dixyrazine or prochlorperazine users (2) Other antipsychotics
Lin et al.	Antipsychotics prescribed for > 30 days during pregnancy (further detail not provided)	Both	Not reported	Random selection (excluded history of chronic illness, including mental illness)	LBW < 2,500g Preterm < 37 weeks	Maternal age, educational level, marital status, hypertension & diabetes, infant's gender and parity, family income, paternal educational level, parental age difference.	**Main analysis:** multivariate logistic regression comparing women with schizophrenia vs women without schizophrenia
Boden et al.	ATC N05A excluding prochlorperazine, levomeproromazine, melperone, lithium	Both	From LMP to parturition.	Women with no antipsychotics dispensed during pregnancy	Preterm < 37 weeks	Maternal age, smoking, maternal country of origin, BMI, cohabitation status, birth order of the infant	**Main analysis:** univariate logistic regression. Multivariate analysis
Habermann et al.	All antipsychotics	Both	Conception (2 weeks gestation) and delivery. Major malformations limited to 1^st^ trimester.	Women unexposed to teratogenic or insufficiently studied medicines.	Congenital malformations classified by 3 experts into major or minor (Genetic syndromes excluded). Preterm < 37 weeks.	Maternal age, alcohol, smoking, BMI, previous spontaneous abortions/previous malformed children, gestation at delivery	**Main analysis:** logistic regression with adjustment for identified confounders.
Sadowski et al.	Minimum of 4 weeks exposure. Women exposed to non-psychiatric known teratogens or substance abuse excluded.	SGA	Not reported.	Exposure to known teratogen/history of psychiatric illness excluded.	Follow-up phone interviews in 2009 - 2012 with participants, with information from infant’s physician (if consented).	Smoking, alcohol, maternal weight, medical history (final risk estimates of interest do not appear to be adjusted**)**	**Main analysis**: Atypical compared to matched unexposed controls. **Second analysis**: Mono atypical vs poly therapy. Pearson’s χ^2^ or Fisher’s exact tests. Multiple linear & logistic regression models Matched for age +/- 3 years, pregnancy duration at initial contact
Bellet et al.	Aripiprazole	SGA	4-10 weeks after LMP (included estimated period of excretion of 28 days)	Pregnant women without exposure or exposed to non-teratogenic agents	Initial standardised phone interview, questionnaire 2 months after birth. Major malformation: EUROCAT classification by 2 experts (chromosomal/clinical genetic syndromes excluded). Preterm birth <37 weeks	Maternal age, smoking, alcohol, gravidity and parity (final risk estimates of interest do not appear to be adjusted**)**	**Main analysis**: Matched for maternal age and gestational age at call 1:2
Sørensen et al.	Antipsychotics (ATCN05A, including lithium)	Both	30 days before estimated conception to one day before outcome. Dichotomized to high and low dose based on DDD.	No antipsychotic during the exposure period. Also compared to women with antipsychotic use before pregnancy (-30 days to -1 year).	Stillbirth >22 weeks (Danish Medical Birth Register)	Psychiatric disease, alcohol/drug abuse, age, cohabitation, income, education, medication	**Main analysis**: binomial regression. Adjusted analysis for stillbirth one covariate at a time due to low numbers of outcome. *Sensitivity analysis*: 1) exposure window 6 months before conception 2) excluding women from unexposed with use 6 months and 30 days before conception 3) ≥ 2 prescriptions; Cox regression with abortion *Subgroups*: Severe mental illness (SA only); DDD (>50% and low ≤50)
Sutter-Dallay et al.	Anti psychotics, antidepressants, mood stabilisers, anxiolytics/hypnotics (exposure from recall/medical records)	Both	Any trimester.	No psychotropic use during pregnancy	Preterm < 36 weeks LBW <2500g	Age, education, parity, presence of partner, maternal diagnosis, smoking	**Main analysis**: Multivariate logistic regression *Sensitivity analysis*: impact of missing data
Vigod et al.	≥2 consecutive antipsychotic prescriptions	Both	Two consecutive antipsychotics between conception and delivery (at least one < 27 weeks), from a drug benefit database	No antipsychotic use during pregnancy	Preterm birth <37 weeks Congenital malformations: ICD 10 CA: Q.00-Q.89 Stillbirth > 20 weeks	Multiple factors in matching including prescription claims, diagnoses and procedures, maternal age	**Main analysis**: High dimensional propensity score (HDPS) matched cohort study (1:1). Poisson regression. *Subgroup*: Atypical and individual. Sensitivity analysis: Trimester *Secondary outcomes*: congenital malformations
Cohen et al.	SGAs	SGA	First trimester	Mostly pregnant women with a history of psychiatric illnesses not treated with SGAs.	Records of major malformations classified by dysmorphologist.	Smoking, alcohol, illicit drugs, psychiatric illness, medication, education, marital status, ethnicity (primary risk estimate unadjusted)	**Main analysis:** unadjusted logistic regression. **Sensitivity analysis:** adjustment to assess confounding, including propensity scores, excluding teratogen exposure
Huybrechts et al.	≥1 antipsychotic	Both	First 90 days of pregnancy	No antipsychotic prescription filled 3 months before start of pregnancy or during 1^st^ trimester	ICD-9 codes from inpatient, outpatient, and procedure records Chromosomal abnormalities excluded.	Calendar year, age, race, smoking, multiple gestation, indication, morbidity, medication, general markers of the burden of illness and morbidity (including alcohol abuse)	**Main analysis:** Generalised linear models with fine stratification on the propensity score. *Subgroups* atypical, typical, and individual Sensitivity analyses: extended propensity score adjustments. Restricted by indication. ≥2 prescriptions. Infant claims only. Dose. Terminations. Exploratory: continuers. Adjusted prescription gap.
Petersen et al.	Antipsychotics in BNF chapter 4.2.1 (excluded prochlorperazine)	Both	Group A) 2 years before *and* between 31-105 days after the start of pregnancy (adverse birth outcomes - last trimester)	Group B) Discontinued: antipsychotic treatment in 2 years before pregnancy but none after 4 weeks before pregnancy Group C) Unexposed: no antipsychotic treatment 2 years before or during pregnancy	Major congenital malformation not defined. Preterm/LBW combined.	Age & calendar year at delivery, BMI≥30 in the year before LMP, illicit drug use, alcohol abuse, smoking, pre-existing medical conditions, concomitant medication listed in BNF chapter 4	**Main analysis**: Poisson regression. Stratified analysis: Atypical vs typical Final model adjusted for age, obesity, alcohol problems, smoking, illicit drug use, and antidepressant prescribing and anticonvulsant mood stabilisers.
Frayne et al.	Antipsychotics, antidepressants or both.	Both	Third trimester	No psychotropic meds	Special care admission discharge summary (preterm not adjusted). Proportion of NICU admissions provided, but adjusted risk not provided. In utero deaths excluded.	BMI, comorbidities, antenatal complication (final risk estimates of interest do not appear to be adjusted**)**	**Main analysis**: Bivariate regression (covariates in adjusted model in article)
Cohen et al.	Quetiapine	SGA	<13 weeks gestation	Women with psychiatric illness without atypical antipsychotic use during pregnancy	Major malformations	Multiple (none included final model)	**Main analysis**: Unconditional logistic regression. Covariates added stepwise individually.
Anderson et al.	SGA	SGA	One month before pregnancy until end of 1^st^ trimester	Controls: liveborn infants without major birth defects who were randomly sampled from the same geographic location	Cases medically reviewed. Single-gene disorders or chromosomal abnormalities excluded.	Multiple (final results unadjusted)	Main analysis: logistic regression Assessed covariates separately for cases and control Maternal diabetes, anticonvulsant exposure and categories <3 cases excluded.
Ellfolk et al.	SGA	SGA (FGA second comparison group)	Purchase of medicine of interest any time during pregnancy or one month before pregnancy.	Unexposed: No antipsychotics from 1 months before pregnancy until end of pregnancy (randomly selected 5:1, matched on year of birth). FGA: during same period (excluding prochlorperazine).	Preterm 32-36 weeks Very preterm <32 weeks. NCU – neonatal care or neonatal intensive care.	Adjusted for year of delivery, age, SES, medication & known teratogens, smoking, pregestational diabetes	**Main analysis**: logistic regression Unexposed controls matched by year of birth to exposed at ratio 1:5. *Subgroup*: “continuous” users – use in more than one trimester
Ellfolk et al.	SGA	SGA (FGA second comparison group)	Purchase of SGA one month before pregnancy until end of 1^st^ trimester	*Unexposed*: No antipsychotics from 3 months before pregnancy until end of first trimester (randomly selected 5:1, matched on year of birth of child). *FGA*: purchase of FGA one month before until end of 1^st^ trimester (prochlorperazine excluded).	Major congenital malformations: ICD-9 (genetic conditions excluded)	Age, parity, BMI (partly), smoking, cohabitation, SES, comorbidities, medication.	**Main analysis**: Logistic regression. Imputation for missingness in covariates. Included covariates with association p<0.1
Freeman et al.	Aripiprazole	SGA	< 13 weeks	Unexposed to atypical antipsychotics in pregnancy	Major malformations (chromosomal and single gene abnormalities excluded)	Age, ethnicity, BMI, alcohol, smoking, illicit drugs, prenatal vitamins, 1^st^ trimester psychotropic medication, primary diagnosis, severity of illness	**Main analysis**: Multivariable logistic regression Final model appears to be adjusted for whether pregnancy planned, smoking, primary diagnosis of bipolar or anxiety.
Viguera et al.	SGA	SGA	< 13 weeks	Unexposed to atypical antipsychotics in pregnancy	Major malformations (chromosomal and single gene abnormalities excluded)	Age, ethnicity, BMI, alcohol, smoking, illicit drugs, prenatal vitamins, 1^st^ trimester psychotropic meds, primary diagnosis, severity of illness	**Main analysis**: Multivariable logistic regression Final model appears to be adjusted for smoking, primary diagnosis of depression or anxiety, SSRI use, prenatal vitamin use
Wang et al.	Antipsychotics listed in BNF chapter 4.2.1	Both	Any use during pregnancy	No antipsychotic use during pregnancy	Preterm birth <37 weeks	Age, year, birth hospital, infant’s sex, parity, comorbidities, SES	**Main analysis**: Cox regression and logistic regression, propensity score fine stratification. *Other*: Comparison with previous exposure; sibling-matched Subgroups: Atypical vs typical, gender, delivery *Sensitivity analysis*: ≥2 prescription, 1/2 weeks prescription extensions, limiting to first pregnancy, excluding 1^st^ pregnancy
Heinonen et al.	Antipsychotics N05A (excluding prochlorperazine, dixyrazine, melperone, lithium)	Both	Any exposure: during pregnancy or 1 month before Early pregnancy: 1 month before and during pregnancy, excluding last 90 days.	1) No antipsychotic use 2) antipsychotic use before or after pregnancy (n = 34,492)	ICD-10 codes	Age, parity, smoking, BMI, use of other neurotropic drugs, caesarean section	Main analysis: Poisson regression in multivariable regression models - any use vs. unexposed. *Other*: antipsychotic groups vs unexposed; early & late pregnancy exposure *Sensitivity analyses*: adjusted for gestation and birth weight Missing data in covariate replaced by overall means
Lin et al.	≥1 antipsychotic (N05A) with a psychiatric indication (excluding lithium, prochlorperazine, levomepromazine)	Both	First 20 weeks of pregnancy	Non-users	Preterm birth <37 weeks LBW <2.5kg	Demographics, comorbid conditions; family history of diabetes, medication, alcohol & substance abuse, frequency of mental health visits	**Main analysis:** Conditional logistic regression and Cox proportional hazards models, with propensity score methods, including matching and inverse probability of treatment weighting. *Subgroups*: Atypical vs. typical *Sensitivity analysis*: restricted to psychiatric indication, high low risk metabolic effects
Yakuwa et al.	≥1 atypical antipsychotics	SGA	4 weeks to 13 weeks after LMP	Women exposed to medicines not known to be teratogenic	Malformations were confirmed by paediatrician’s report (EUROCAT).	Smoking, alcohol, other comorbidities	Main analysis: Logistic regression and inverse probability weighting (IPW) to adjust for confounding factors. Other: elective abortion
Cohen et al.	Lurasidone and quetiapine	SGA	≤13 weeks	Unexposed to SGAs during pregnancy	Major malformation (chromosomal and single gene abnormalities excluded)	Age, ethnicity, BMI, alcohol, smoking, illicit drugs, prenatal vitamins, 1^st^ trimester psychotropic meds, primary diagnosis, severity of illness	**Main analysis**: Unadjusted odds ratios calculated and as sensitivity analysis variates added individually
Huybrechts et al.	≥1 antipsychotic prescription (excluding prochlorperazine)	Both	1^st^ trimester	No antipsychotic prescriptions during the 3 months before pregnancy and during 1^st^ trimester	Major congenital malformations, and specific malformation subtypes previously associated with antipsychotics Chromosomal abnormality excluded.	Demographic factors, indication, comorbidities, lifestyle behaviours, meds, healthcare utilisation	**Main analysis**: Propensity score fine stratification with weighting approach. *Sensitivity analyses*: ≥2 prescriptions; only with psychiatric indication; drug/class monotherapy
Kananen et al.	ATC N05A excluding lithium (self-reported or records of antipsychotic use in hospital)	Both	Not clearly defined.	No antipsychotic	Preterm delivery: <37 weeks. Congenital malformations or chromosomal abnormalities ICD-10 codes in Q category	Age, BMI, smoking, alcohol, illicit drugs, comorbidities, other psychotropic medicine	**Main analysis**: Logistic and linear regression (excluded multifetal & hyperemesis migraine indication) *Sensitivity analyses*: Included hyperemesis indication.
Liu et al.	ATC N05A (excluding lithium) from 30 days before pregnancy to 84 days.	Both	1^st^ trimester	No antipsychotic prescription in 1^st^ trimester.	Major malformations according to EUROCAT malformation classification (prenatal and up to 1 year after birth) Fetal chromosomal abnormalities, and coding errors excluded.	Ethnicity, age at index pregnancy, primiparity, education, pre-pregnancy BMI, smoking, marital status, psychiatric diagnosis at start of pregnancy, hospital psychiatric contact within one-year, other medication, calendar years	**Main analysis:** Log-binomial regression to estimate adjusted prevalence ratio and propensity score fine stratifications. *Subgroups:* Atypical vs typical, 5 most common individually. *Sensitivity analyses:* Discontinuers (antipsychotics 2 years to 1 month before pregnancy); sibling control; ≥2 prescriptions; live births only
Viguera et al.	Olanzapine	SGA	1^st^ trimester	Unexposed to SGAs during pregnancy	Major malformation (chromosomal and single gene abnormalities excluded)	Age, ethnicity, BMI, alcohol, smoking, illicit drugs, prenatal vitamins, 1^st^ trimester psychotropic medication, primary diagnosis, severity of illness	Not described - zero outcome events in main exposure of interest

When study samples overlapped for the same outcomes, the study with the largest sample size for the relevant estimates was preferentially included in the meta-analysis. A total of ten studies were excluded due to overlapping data sources. Eight were excluded as they had a smaller sample size (Bodén et al. 2012; Cohen et al. 2016, 2018, 2023; Huybrechts et al. 2016; Freeman et al. 2021; Liu et al. 2023; Viguera et al. 2023). Two (Ellfolk et al. 2021; Heinonen et al. 2022) were excluded as they had a similar sample size with another multi-country study (Huybrechts et al. 2023), but the latter study did not report estimates by individual country, so the overall estimates from this study were used.

Ten studies were excluded from the meta-analysis based on their Newcastle-Ottawa Scale (NOS) score. Eight of these studies did not make statistical adjustments to control for potential confounding factors in the risk estimates of interest, and hence did not score in the comparability domain (Rumeau-Rouquette et al. 1977; Slone et al. 1977; McKenna et al. 2005; Sadowski et al. 2013; Bellet et al. 2015; Sørensen et al. 2015; Frayne et al. 2017; Anderson et al. 2020). The remaining two studies were excluded as they scored less than 6 overall (Diav-Citrin et al. 2005; Sutter-Dallay et al. 2015). Newcastle-Ottawa Scale scores for all studies are provided in the supplementary information.

Twelve studies (comprising over 10 million pregnancies across 12 countries) met the inclusion criteria for the meta-analysis. Adjusted risk estimates or studies with matched samples were extracted (supplementary information). For studies that did not report antipsychotics as a single exposure group, the exposure group with the larger sample size (SGA or FGA) was used as the antipsychotic exposure group (Habermann et al. 2013; Ellfolk et al. 2020; Huybrechts et al. 2023). Lin et al. (2010) provided adjusted risk estimates with untreated pregnant women with schizophrenia as the reference group for the adjusted results. Given that the adjusted and crude risk estimates were similar for preterm birth, the odds ratios and confidence intervals were calculated using all unexposed women as the reference group (that is both women without schizophrenia and women with schizophrenia who had not been prescribed antipsychotics during the exposure period). Reis and Källén (2008) provided separate risk estimates for antipsychotics primarily used as anti-nausea treatment (dixyrazine/prochlorperazine) and antipsychotics excluding these medications. The risk estimates for the latter group were included in the main analysis, with a pooled estimate included in a sensitivity analysis.

### Congenital malformations

**Fig. 2:**
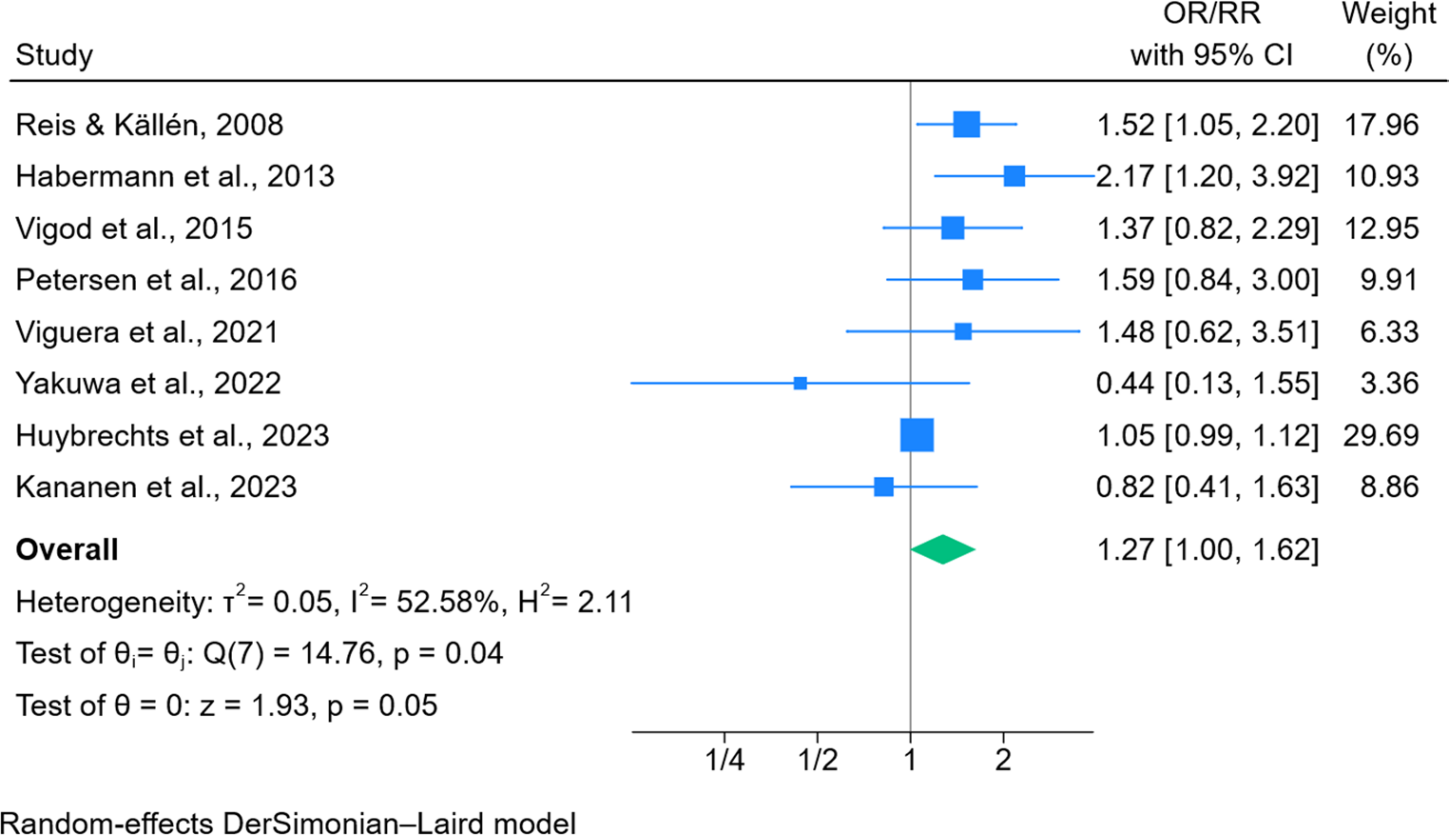
Forest plot of antipsychotic exposure and congenital malformations

Risk estimates for major or relatively severe congenital malformations were prioritised in the analysis. Five studies reported adjusted odds ratios (Reis and Källén 2008; Habermann et al. 2013; Viguera et al. 2021; Yakuwa et al. 2022; Kananen et al. 2023) and three reported adjusted relative risks (Vigod et al. 2015; Petersen et al. 2016; Huybrechts et al. 2023). In total, eight studies were included in the meta-analysis of congenital malformation (Fig. 2). The pooled results indicated borderline evidence of an increase in the odds of congenital malformations following in utero antipsychotic exposure (OR 1.27; 95% CI 0.996–1.624, *p* = 0.0535). There was evidence of moderate heterogeneity among the studies included (I² = 52.6%), with the Q-test indicating marginal evidence of heterogeneity (*p* = 0.04), suggesting the presence of some variability across studies.

A leave-one-out meta-analysis demonstrated that exclusion of any individual study resulted in odds ratios ranging from 1.18 to 1.39, with lower confidence intervals falling below 1.00 when excluding any of the studies published before 2022 (Fig. 5, supplementary material). A subgroup analysis categorised studies by the location of the data source (Fig. 6, supplementary material). Most studies utilised Europe or North American data sources. Yakuwa et al. (2022) was the only study to use data from Asia, and provided the smallest weighting to the overall estimate (3.4%).

### Sensitivity analyses: congenital malformations

Two sensitivity analyses were conducted, neither reached the conventional threshold for statistical significance. The first excluded studies that included chromosomal abnormalities in the outcome of congenital malformations and that did not limit the exposure period during pregnancy to the first trimester (Reis and Källén 2008; Kananen et al. 2023). This resulted in a pooled odds ratio of 1.30 (95% CI 0.95–1.77, *p* = 0.06, Fig. 7, supplementary information). A second sensitivity analysis applied the estimates for Reis and Källén (2008) used in the meta-analysis by Wang, Brauer, et al. (2021). These were calculated after pooling results for antipsychotic vs. unexposed results (i.e. dixyrazine/prochlorperazine vs. unexposed, and other antipsychotics vs. unexposed). This adjustment attenuated the pooled odds ratio to 1.20 (95% CI 0.94–1.52, *p* = 0.14, Fig. 8, supplementary information).

### Second-generation antipsychotics and congenital malformations

**Fig. 3:**
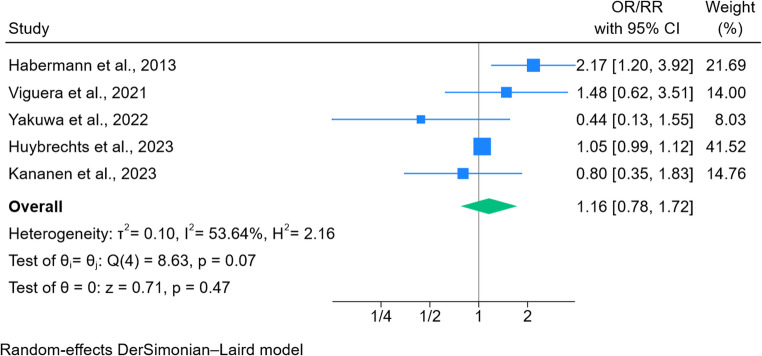
Forest plot of second-generation antipsychotic exposure and congenital malformations

When restricting the analysis to second-generation antipsychotics, the pooled odds ratio was 1.16 (95% CI 0.78–1.72, *p* = 0.47, Fig. 3). Moderate heterogeneity was observed (I² = 54%). The study by Huybrechts et al. (2023) contributing the largest weight (41.5%). A leave-one-out analysis did not substantially change the estimates (Fig. 9, supplementary information).

### Preterm birth

**Fig. 4:**
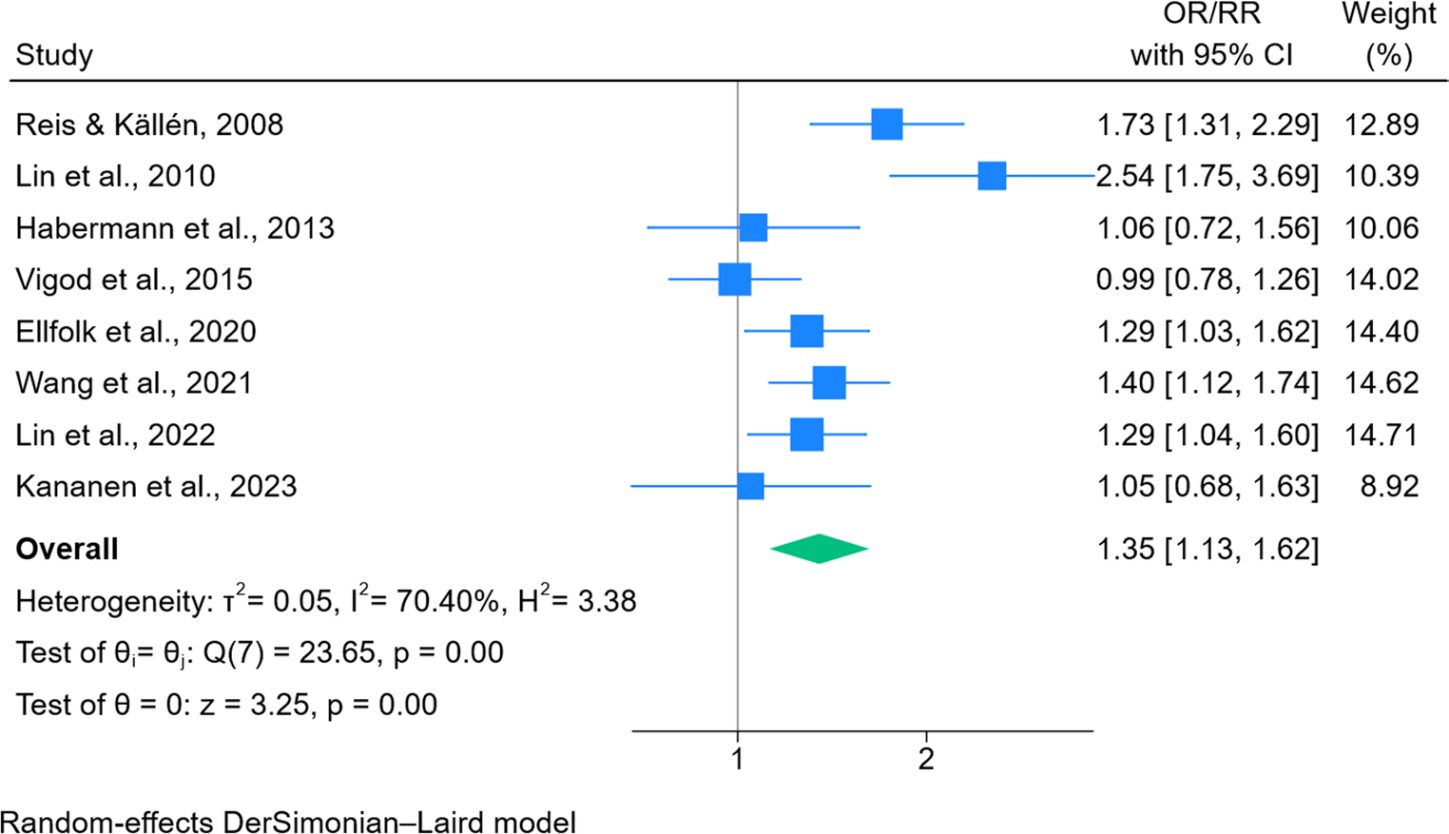
Forest plot of association between in utero antipsychotic exposure and preterm birth

Eight studies met the criteria for inclusion in the meta-analysis (Fig. 4). Ellfolk et al. (2020) provided adjusted risk estimates for the outcome of preterm birth (32–36 weeks) and very preterm births (< 32 weeks), the latter was included in the meta-analysis. The pooled odds ratio for preterm birth following antipsychotic exposure was 1.35 (95% CI 1.13–1.62, *p* = 0.0012). Studies published since 2020 represented over 50% of the weight. Of note, moderate to high evidence of heterogeneity was present (I^2^ = 70.4%), with the Q-test indicating statistical significance (*p* < 0.05). A leave-one-out analysis did not substantially change the results (Fig. 10, supplementary information).

### Other outcomes and exposures

Due to limited data, the outcomes of low birth weight, stillbirth, and NICU admission (each assessed in three or fewer studies) were not included in the meta-analysis. Similarly, analyses of first-generation antipsychotics or individual second-generation antipsychotics and the outcome of congenital abnormality were not conducted.

### Publication bias

An assessment for publication bias was conducted for studies investigating congenital malformations. Visual inspection of the funnel plot did not reveal strong evidence of publication bias (Fig. 11 supplementary information).

## Discussion

The analysis of risk of congenital malformations following in utero antipsychotic exposure did not indicate strong evidence of a substantial increase in teratogenic risk. When results from eight studies were pooled, evidence of an association was borderline (OR 1.27; 95% CI 0.996–1.624, *p* = 0.0535) and did not reach the conventional threshold for statistical significance (i.e. the confidence interval included the null). The overall pooled risk estimate was similar across two sensitivity analyses, with neither reaching the conventional threshold for statistical significance. When limiting to risk estimates reporting exposure to second-generation antipsychotics, we did not find evidence of an association with congenital malformations (OR 1.16; 95% CI 0.78–1.72, *p* = 0.47).

In comparison to other meta-analyses, the overall risk estimate for congenital malformations is similar to the meta-analysis conducted by Wang, Brauer, et al. (2021) who reported a pooled adjusted risk ratio of 1.23 (95% CI 0.96–1.58). We included estimates from an additional three studies in the meta-analysis that have been published since their review. In terms of differences, we included a risk estimate from the study by Vigod et al. (2015) that was limited to first trimester exposure, we also included the risk estimate from exposure to antipsychotics other than dixyrazine/prochlorperazine in the main analysis, but included a pooled estimate in one of the sensitivity analyses. Wang, Brauer, et al. (2021) reported a pooled risk estimate of 1.35 (95% CI 0.73–2.47) following second-generation antipsychotic exposure based on three studies, we included results from five studies. Coughlin et al. (2015) reported a pooled odds ratio of 2.12 (95% CI 1.25–3.57) after antipsychotic exposure based on crude estimates and did not consider the risk of bias with individual studies. Wang et al. (2025) performed a network meta-analysis of 22 studies, reporting an increased risk of congenital malformations with quetiapine [odds ratio (OR), 1.19; 95% credible interval (CrI), 1.01–1.39], aripiprazole (OR, 1.30; 95% CrI 1.10–1.65), olanzapine (OR, 1.33; 95% CrI 1.11–1.64), and risperidone (OR, 1.43; 95% CrI 1.18–1.77), a statistically significant association was not observed for ziprasidone or haloperidol. Thirteen of the studies were considered to be at high/serious risk of bias, highlighting the need for caution interpreting the findings.

Chan et al. (2024) published a cohort study using data from Hong Kong after our search end date. Although they did not report risks associated with overall antipsychotic use, they observed an increased risk of major congenital malformations following in utero SGA exposure (aOR 2.11; 95% CI 1.19–3.86) but not FGA exposure (aOR 1.63; 95% CI 0.86–3.19). The authors acknowledged the potential for residual confounding as they were unable to adjust for socioeconomic status or tobacco use.

For preterm birth, antipsychotic exposure was associated with an increased odds ratio (OR 1.35; 95% CI 1.13–1.62, *p* < 0.0012), though heterogeneity was moderate to high across studies. Two previous meta-analysis also reported increased odds ratios. In comparison to this meta-analysis, both included crude estimates in the main analysis and did not exclude studies considered to be at high risk of bias. Coughlin et al. (2015) reported a pooled odds ratio of 1.86 (95% CI 1.45–2.39) based on 7 studies. More recently Joseph-Delaffon et al. (2024) reported a pooled odds ratio of 1.67 (95% CI 1.25–2.24, I^2^ = 79%) following FGA exposure, and a pooled odds ratio of 1.54 (95% CI 1.24–1.92, I^2^ = 64%) following SGA exposure (including both unadjusted and adjusted risk estimates).

Maternal psychiatric illness has been associated with adverse birth outcomes (Jablensky et al. 2005; Heun-Johnson et al. 2019; Mongan et al. 2019; Howard and Khalifeh 2020). Many studies in this analysis did not control for the severity of illness or treatment indication. However, more recently published studies have used additional methods to attempt to control for confounding by indication. For example, Liu et al. (2023) reported that risk estimates for congenital malformations were attenuated with an adjusted prevalence ratio of 1.14 (95% CI 0.88–1.48) when comparing antipsychotic exposure vs. discontinuers and a sibling analysis estimate of 1.08 (95% CI 0.47–2.49). Similarly, Wang, Chan, et al. (2021) reported attenuated estimates for preterm birth when the infants of women who had taken antipsychotics during pregnancy were compared to antipsychotic exposure before pregnancy (weighted OR 0.93; 95% CI 0.70–1.24), and also in a sibling-matched analysis (weighted OR 1.25; 95% CI 0.85–1.82).

Substance use, including tobacco and alcohol use, has been linked to several adverse outcomes in infants (Louw 2018; Chang 2020; Steele et al. 2020). Baseline rates were reported to differ between women who were prescribed antipsychotics during pregnancy compared to women who were not. Before propensity-score weighting, the proportion of women with evidence of tobacco use during pregnancy in the study by Huybrechts et al. (2023) was 31% in the exposed cohort vs. 11% compared to unexposed cohort in the Nordic countries; and 13% vs. 4% in the U.S. cohorts. Similarly, Kananen et al. (2023) reported increased baseline proportions of tobacco use (21.5 vs. 7.6%), alcohol use (4.9 vs. 0.5%), and illicit drug use (7.7% vs. 0.4%).

Not all data sources have available information on potentially relevant confounders, for example Reis and Källén (2008) did not include alcohol use or alcohol abuse as a covariate. Socioeconomic status has also been found to have an impact on birth outcomes, including low birth weight (Thomson et al. 2021). Some studies included in the low birth weight meta-analysis did not include a measure of socioeconomic status as a covariate (Reis and Källén 2008; Lin et al. 2022; Kananen et al. 2023).

Studies varied in how they defined exposure groups. For example, the first-generation antipsychotic prochlorperazine is used to varying degrees in different countries for pregnancy-related nausea (Reutfors et al. 2020; Fisher et al. 2022); some studies that investigated the risk of congenital malformations did not include prochlorperazine in the antipsychotic exposed group (Petersen et al. 2016; Huybrechts et al. 2023),

Studies also differed in how they accounted for maternal exposure to other medication. As an example, studies have reported differences in the proportion of anticonvulsant usage in the antipsychotic exposed group compared to the unexposed group, some anticonvulsants are known to increase the risk of congenital malformations following in utero exposure (Battino et al. 2024). In the study by Viguera et al. (2021), the proportion of concomitant anticonvulsant use in the exposed group was 34.1% vs. 12.6% in the unexposed. Huybrechts et al. (2023) reported unadjusted baseline anticonvulsant concomitant prescribing of 0.4% in unexposed women vs. 7% in FGA-exposed, and 16% in the SGA-exposed women in the Nordic countries; and 1.6% in unexposed women vs. 17% in FGA-exposed women, and 25% in the SGA-exposed women in the US cohort. There were some differences in how these factors were handled; Reis and Källén (2008) performed an additional analysis excluding anticonvulsant exposed pregnancies, Petersen et al. (2016) included anticonvulsant mood stabilisers in the adjusted model, and Huybrechts et al. (2023) used propensity score weighting.

### Strengths

A strength of this review is that comprehensive literature searches were performed, and it builds upon previous knowledge by including results from more recent large observational studies. Observational studies are subject to limitations, most notably confounding. We extracted only matched or adjusted results to mitigate this limitation. Metelli and Chaimani (2020) acknowledged the challenges of conducting meta-analyses related to extracting data from observational studies and recommended the identification of the most important confounders and extracting results that were adjusted for as many of these confounders as possible. We followed this approach with one exception, the risk of preterm estimates in the study by Lin et al. (2010). The adjusted and unadjusted results were similar, and we calculated the odds ratio to include a baseline group of unexposed women (with and without schizophrenia).

### Limitations

The primary limitation of this review is related to the potential for confounding, despite primarily including adjusted estimates, it is possible that residual confounding remains. There is also a risk of misclassification bias, as most studies using electronic health data are unable to determine whether a woman who has been issued a prescription will have taken the medication. This is particularly relevant during pregnancy, when women might be more reluctant to take a medicine after discovering that they are pregnant. To reduce the risk of misclassification bias, some studies included sensitivity analyses that varied the definition of exposure. We performed a sensitivity analysis by restricting the analysis to studies that limited pregnancy exposure to the first trimester. To avoid double counting, if estimates were not available for antipsychotics overall then estimates for the largest group were employed (SGA or FGA). There is also a risk of outcome misclassification as not all outcomes were validated. Some studies did not have information relating to early miscarriage or elective terminations, and this could result in selection bias. Finally, we were unable to explore the risk associated with different antipsychotics or dose.

## Conclusion

The decision to continue antipsychotic use during pregnancy involves a complex balance of risks and benefits for both women and their healthcare providers. Any potential risks to the developing fetus must be weighed against the risks of discontinuing treatment, including the possibility of relapse in women with severe mental illness, which can have serious consequences for both the woman and her infant. Consistent with previous meta-analyses, we did not find strong evidence that in utero antipsychotic exposure is a major teratogen. We observed an association between maternal antipsychotic use and preterm birth, though there was significant heterogeneity across the studies.

Despite publication of large observational studies in recent years, the evidence remains insufficient to make conclusive decisions regarding the risks of individual antipsychotics. Future research should focus on the association of individual antipsychotics and adverse neonatal outcomes and specific congenital malformations using robust methods to account for potential confounding.

## Supplementary Information

Below is the link to the electronic supplementary material.

Supplementary Material 1 (DOCX 602 KB)

## Data Availability

Not applicable.
