# Supplementary material for: Antipsychotic drug use during pregnancy and neonatal outcomes: a systematic review and meta-analysis
Source: Arch Womens Ment Health. 2026 Jan 8;29(1):12. doi: 10.1007/s00737-025-01651-5 (PMC12783257; doi:10.1007/s00737-025-01651-5)
Supplement: Supplementary file 1 — Supplementary Material 1 (DOCX 602 KB) [file 737_2025_1651_MOESM1_ESM.docx]

**Antipsychotic drug use during pregnancy and neonatal outcomes: a systematic review and meta-analysis – supplementary information**

**Search terms**

| Embase <1974 to 2024 February 21> | | |
| --- | --- | --- |
| 1 | exp Pregnancy/ or exp Maternal Exposure/ or Prenatal Exposure Delayed Effects/ or (maternal or pregnan$).m_titl. | 919494 |
| 2 | neuroleptic agent/ | 92763 |
| 3 | (antipsychotic or chlorpromazine or fluphenazine or levomepromazine or pericyazine or prochlorperazine or promazine or trifluoperazine or benperidol or haloperidol or flupentixol or zuclopenthixol or pimozide or sulpiride or Amisulpride or aripiprazole or asenapine or cariprazine or clozapine or lurasidone or olanzapine or paliperidone or quetiapine or risperidone).tw. | 121305 |
| 4 | exp Pregnancy Outcome/ or apgar score/ or stillbirth/ or fetal death/ or birth weight/ or infant, low birth weight/ or premature birth/ or Intensive Care Units, Neonatal/ or fetus/ or infant, newborn/ | 934856 |
| 5 | (birth outcome* or neonatal outcome*).tw. | 32838 |
| 6 | congenital abnormalities/ or Teratology/ | 17094 |
| 7 | (congenital abnormalit* or deformit* or congenital defect* or birth defect* or malform*).mp. | 528116 |
| 8 | 2 or 3 | 179417 |
| 9 | 4 or 5 or 6 or 7 | 1393480 |
| 10 | 1 and 8 and 9 | 898 |
| 11 | exp animal/ not human/ | 5354699 |
| 12 | 10 not 11 | 789 |
| 13 | limit 12 to english language | 697 |

| Ovid MEDLINE(R) ALL <1946 to February 21, 2024> | | |
| --- | --- | --- |
| 1 | exp Pregnancy/ or exp Maternal Exposure/ or Prenatal Exposure Delayed Effects/ or (maternal or pregnan$).m_titl. | 1090204 |
| 2 | Antipsychotic Agents/ | 60049 |
| 3 | (antipsychotic or chlorpromazine or fluphenazine or levomepromazine or pericyazine or prochlorperazine or promazine or trifluoperazine or benperidol or haloperidol or flupentixol or zuclopenthixol or pimozide or sulpiride or Amisulpride or aripiprazole or asenapine or cariprazine or clozapine or lurasidone or olanzapine or paliperidone or quetiapine or risperidone).tw. | 90158 |
| 4 | exp Pregnancy Outcome/ or apgar score/ or stillbirth/ or fetal death/ or birth weight/ or infant, low birth weight/ or premature birth/ or Intensive Care Units, Neonatal/ or fetus/ or infant, newborn/ | 838691 |
| 5 | (birth outcome* or neonatal outcome*).tw. | 22280 |
| 6 | congenital abnormalities/ or Teratology/ | 35770 |
| 7 | (congenital abnormalit* or deformit* or congenital defect* or birth defect* or malform*).mp. | 275222 |
| 8 | 2 or 3 | 113888 |
| 9 | 4 or 5 or 6 or 7 | 1074787 |
| 10 | 1 and 8 and 9 | 478 |
| 11 | exp animals/ not humans/ | 5197703 |
| 12 | 10 not 11 | 418 |
| 13 | limit 12 to english language | 353 |

| APA PsycInfo <1967 to February Week 3 2024> | | |
| --- | --- | --- |
| 1 | exp Pregnancy/ or exp Prenatal Exposure/ or exp Prenatal Development/ or (maternal or pregnan$).m_titl. | 79588 |
| 2 | neuroleptic drugs/ | 23368 |
| 3 | (antipsychotic or chlorpromazine or fluphenazine or levomepromazine or pericyazine or prochlorperazine or promazine or trifluoperazine or benperidol or haloperidol or flupentixol or zuclopenthixol or pimozide or sulpiride or Amisulpride or aripiprazole or asenapine or cariprazine or clozapine or lurasidone or olanzapine or paliperidone or quetiapine or risperidone).tw. | 47404 |
| 4 | exp Pregnancy Outcome/ or exp birth weight/ or exp Premature Birth/ or exp Neonatal Intensive Care/ or exp Fetus/ or exp Birth/ | 27806 |
| 5 | (birth outcome* or neonatal outcome*).tw. | 2227 |
| 6 | exp Congenital Disorders/ or Teratology/ | 8929 |
| 7 | (congenital abnormalit* or deformit* or congenital defect* or birth defect* or malform* or apgar* or stillbirth or fetal death).mp. | 8883 |
| 8 | 2 or 3 | 52940 |
| 9 | 4 or 5 or 6 or 7 | 43621 |
| 10 | 1 and 8 and 9 | 135 |
| 11 | (animal not human).po. | 380624 |
| 12 | 10 not 11 | 130 |

**Tables of Newcastle-Ottawa scores of cohort and case control studies**

| Authors | Year | Selection | | | | Comparability | | Outcome | | | Total |
| --- | --- | --- | --- | --- | --- | --- | --- | --- | --- | --- | --- |
|  |  | Representativeness  of the exposed cohort | Selection of  the  nonexposed  cohort | Ascertainment  of exposure | Demonstration that outcome of interest was not present at the start of the study | Study  controls  for maternal  age,  smoking,  alcohol  consumption | Study  controls  for any  additional  factor | Assessment  of outcome | Was follow-up long enough  for outcomes  to occur? | Adequacy  of follow  up of  cohorts |  |
| Slone et al. | 1977 | 1 | 1 | 1 | 1 | 0 | 0 | 0 | 0 | 0 | 4 |
| Rumeau-Rouquette et al. | 1977 | 1 | 1 | 1 | 1 | 0 | 0 | 1 | 1 | 1 | 7 |
| Diav-Citrin et al. | 2005 | 0 | 1 | 1 | 1 | 1 | 0 | 0 | 1 | 0 | 5 |
| McKenna et al. | 2005 | 0 | 0 | 1 | 1 | 0 | 0 | 1 | 1 | 0 | 4 |
| Reis et al. | 2008 | 1 | 1 | 1 | 1 | 0 | 1 | 1 | 1 | 1 | 8 |
| Lin et al. | 2010 | 1 | 1 | 1 | 1 | 0 | 1 | 1 | 1 | 1 | 8 |
| Habermann et al. | 2013 | 0 | 1 | 1 | 1 | 1 | 1 | 1 | 1 | 1 | 8 |
| Sadowski et al. | 2013 | 0 | 0 | 0 | 1 | 0 | 0 | 1 | 1 | 0 | 3 |
| Bellet et al. | 2015 | 0 | 1 | 1 | 1 | 0 | 0 | 1 | 1 | 0 | 5 |
| Sørensen et al. | 2015 | 1 | 1 | 1 | 1 | 0 | 0 | 1 | 1 | 1 | 7 |
| Sutter-Dallay et al. | 2015 | 0 | 1 | 0 | 1 | 0 | 1 | 0 | 1 | 1 | 5 |
| Vigod et al. | 2015 | 1 | 1 | 1 | 1 | 0 | 1 | 1 | 1 | 1 | 8 |
| Petersen et al. | 2016 | 1 | 1 | 1 | 1 | 1 | 1 | 1 | 1 | 1 | 9 |
| Frayne et al. | 2017 | 0 | 1 | 1 | 1 | 0 | 0 | 1 | 1 | 1 | 6 |
| Ellfolk et al. | 2020 | 1 | 1 | 1 | 1 | 0 | 1 | 1 | 1 | 1 | 8 |
| Viguera et al. | 2021 | 0 | 1 | 1 | 1 | 1 | 1 | 1 | 1 | 0 | 7 |
| Wang, et al. | 2021 | 1 | 1 | 1 | 1 | 0 | 1 | 1 | 1 | 1 | 8 |
| Lin et al. | 2022 | 1 | 1 | 1 | 1 | 0 | 1 | 1 | 1 | 1 | 8 |
| Yakuwa et al. | 2022 | 0 | 1 | 1 | 1 | 1 | 1 | 0 | 1 | 1 | 7 |
| Huybrechts et al. | 2023 | 1 | 1 | 1 | 1 | 1 | 1 | 1 | 1 | 1 | 9 |
| Kananen et al. | 2023 | 1 | 1 | 0 | 1 | 1 | 1 | 1 | 1 | 1 | 8 |

**Table 2: Newcastle-Ottawa scores of cohort studies**

| Authors | Year | Case definition | Representativeness | Selection of controls | Definition of controls | Study  controls  for maternal  age, smoking,  alcohol  consumption | Study  controls  for any  additional  factor | Ascertainment | Same method of ascertainment for cases and controls | Non-response rate |  |
| --- | --- | --- | --- | --- | --- | --- | --- | --- | --- | --- | --- |
| Anderson et al. | 2020 | 1 | 1 | 1 | 1 | 0 | 0 | 0 | 1 | 1 | 6 |

**Table 3: Newcastle-Ottawa scores of case control studies**

**Table 4: Adjusted risk estimates from included studies**

| Reference | Risk estimates | |
| --- | --- | --- |
|  | Congenital malformation | Preterm |
| Reis et al. 2008 | Relatively severe congenital malformations:  Dixyrazine or prochlorperazine vs. population: aOR 0.67 (0.49 – 0.90)  Other antipsychotic vs. population**:** aOR 1.52 (1.05 – 2.19) | Dixyrazine or prochlorperazine vs. population: aOR 1.04 (0.86 – 1.27)  Other antipsychotic vs. population**:** aOR 1.73 (1.31–2.29) |
| Lin et al.  2010 |  | FGA vs. unexposed women with schizophrenia**:**  aOR 2.46 (1.50-4.11)  SGA vs. unexposed women with schizophrenia**:**  aOR 1.61 (0.63-4.12)  (calculated antipsychotics vs. unexposed OR 2.54 (1.73 – 3.66) |
| Habermann et al.  2013 | Major malformations:  FGA vs unexposed: aOR 1.71 (0.78 – 3.76)  SGA vs unexposed: aOR 2.17 (1.20 -3.91) | FGA vs unexposed: OR 1.96 (1.29 -2.98)  SGA vs unexposed: OR 1.06 (0.72 – 1.56) |
| Vigod et al.  2015 | Congenital malformations:  Exposed vs unexposed: aRR 1.37 (0.82 – 2.29) | Exposed vs unexposed: aRR: 0.99 (0.78 to 1.26) |
| Petersen et al.  2016 | Major congenital malformations:  Exposed vs unexposed: aRR 1.59 (0.84 – 3.00) |  |
| Ellfolk et al.  2020 |  | Preterm 32-36 weeks.  SGA vs unexposed: aOR 1.29 (1.03–1.62) |
| Viguera et al.  2021 | Major malformations:  SGA vs. unexposed: aOR 1.48 (0.625 – 3.517) |  |
| Wang et al.  2021 |  | Exposed vs unexposed: wOR 1.40 (1.13 - 1.75) |
| Lin et al.  2022 |  | Exposed vs. unexposed: aHR 1.29 (1.04 - 1.60) |
| Yakuwa et al.  2022 | Major congenital malformations.  SGA vs. unexposed: aOR 0.44 (0.12 - 1.48) |  |
| Huybrechts et al.  2023 | Major congenital malformation:  SGA vs unexposed: aRR 1.05 (0.98 – 1.11)  FGA vs unexposed: aRR 1.13 (0.98 – 1.29) |  |
| Kananen et al.  2023 | Congenital malformations or chromosomal abnormalities:  Any antipsychotic vs. unexposed: aOR 0.82 (0.41 – 1.63)  Quetiapine vs. unexposed: aOR 0.80 (0.35 – 1.84) | Any antipsychotic vs. unexposed: aOR 1.05 (0.68 – 1.63) |


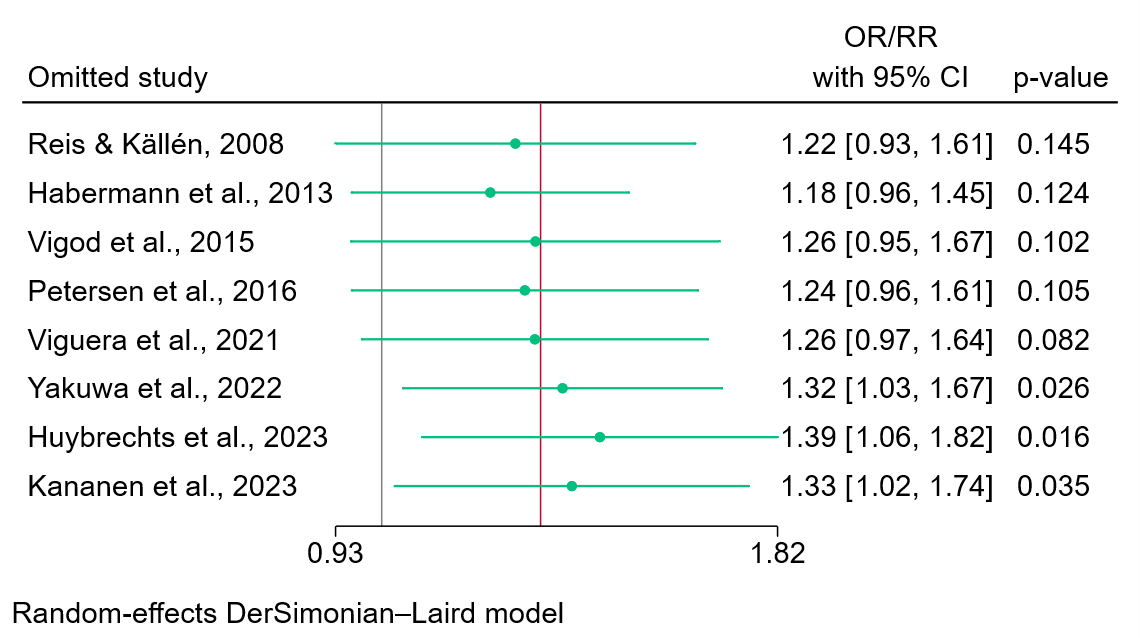


**Figure 5: Leave-one-out analysis of antipsychotic exposure and congenital malformations**


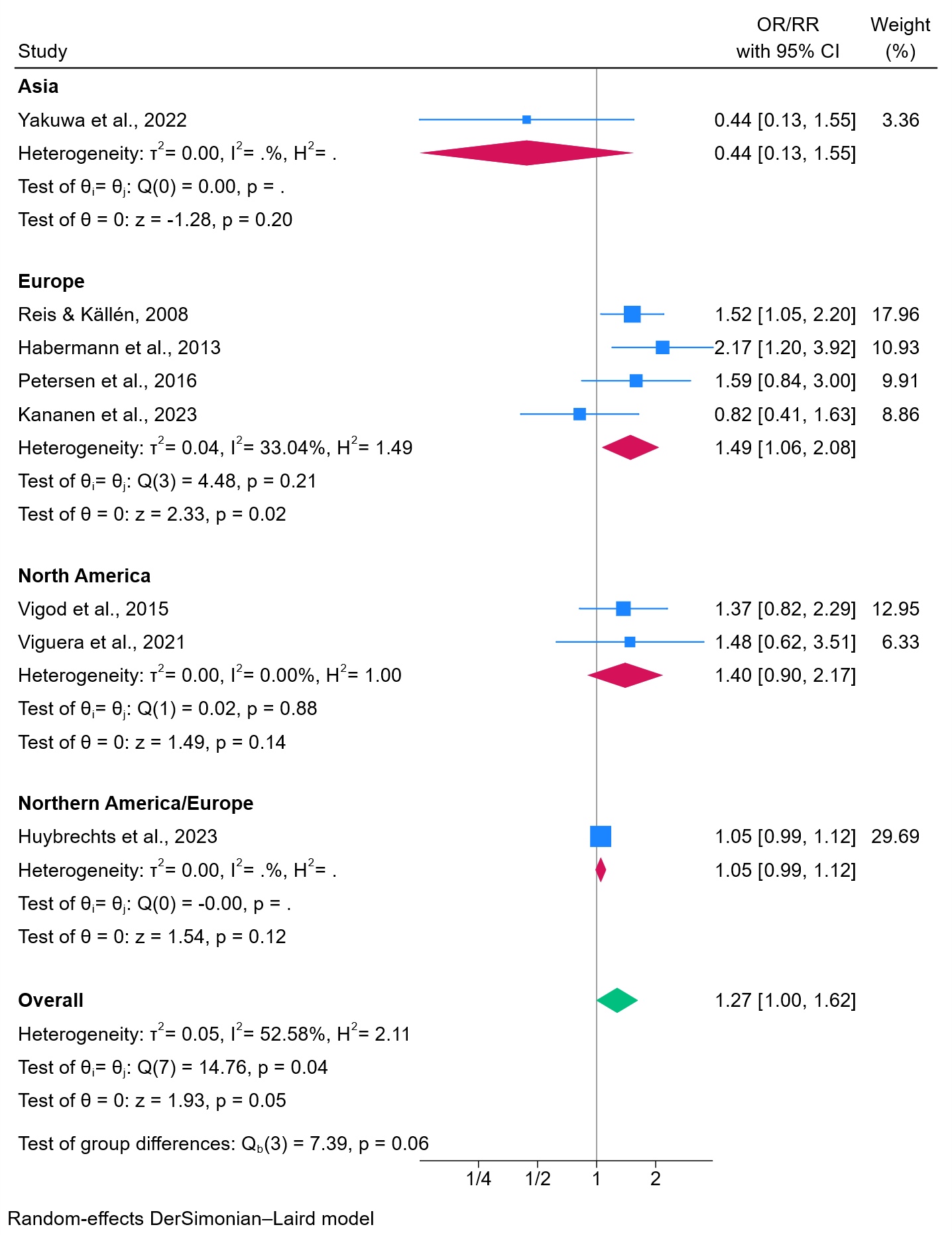


**Figure 6: Antipsychotic exposure and risk of congenital malformations by region**

**Forest plots of sensitivity analyses**


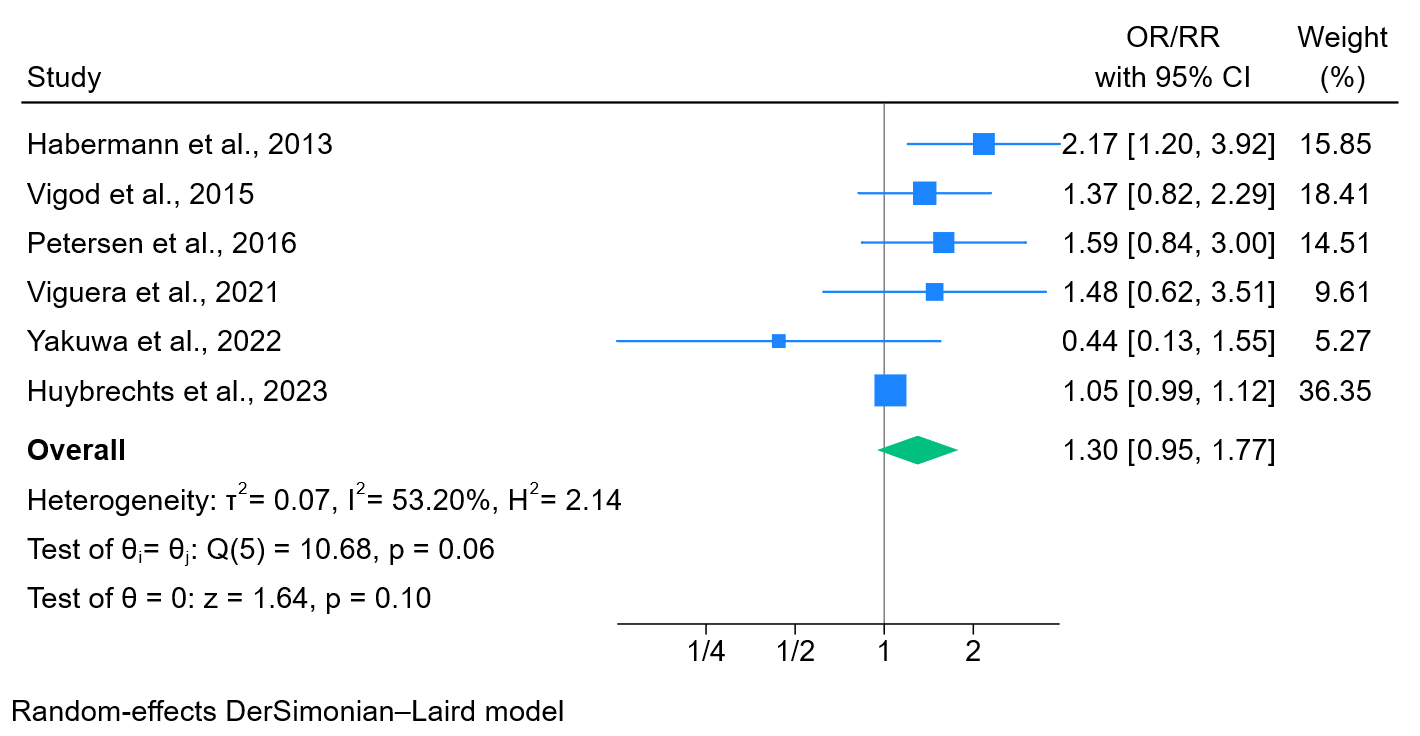


**Figure 7: Forest plot of in utero antipsychotic exposure and congenital malformations excluding studies that included chromosomal abnormalities and that did not limit exposure during pregnancy to the first trimester (sensitivity analysis)**


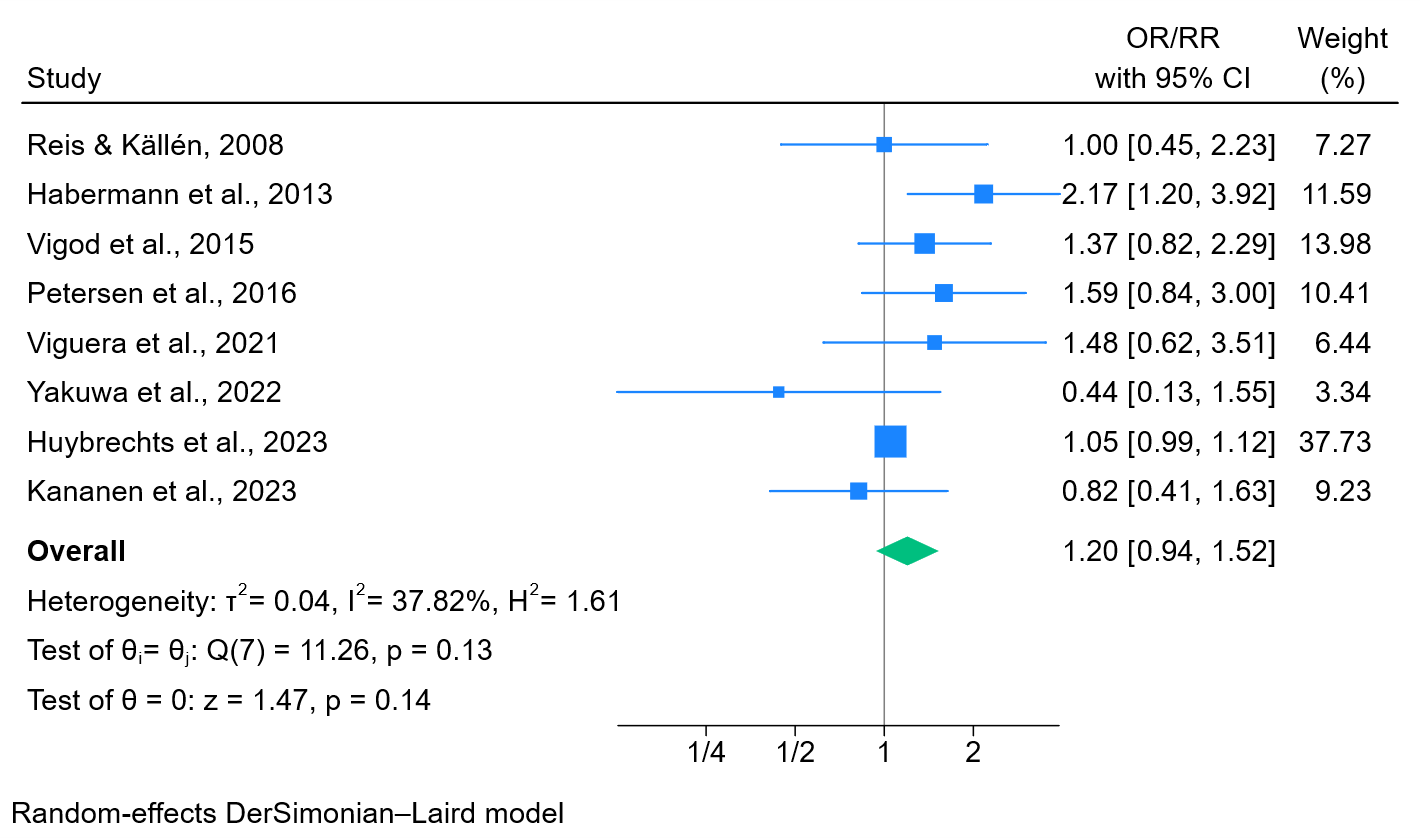


**Figure 8: Forest plot of in utero antipsychotic exposure and congenital malformations with pooled estimates from Reis & Källén (2008) (sensitivity analysis)**


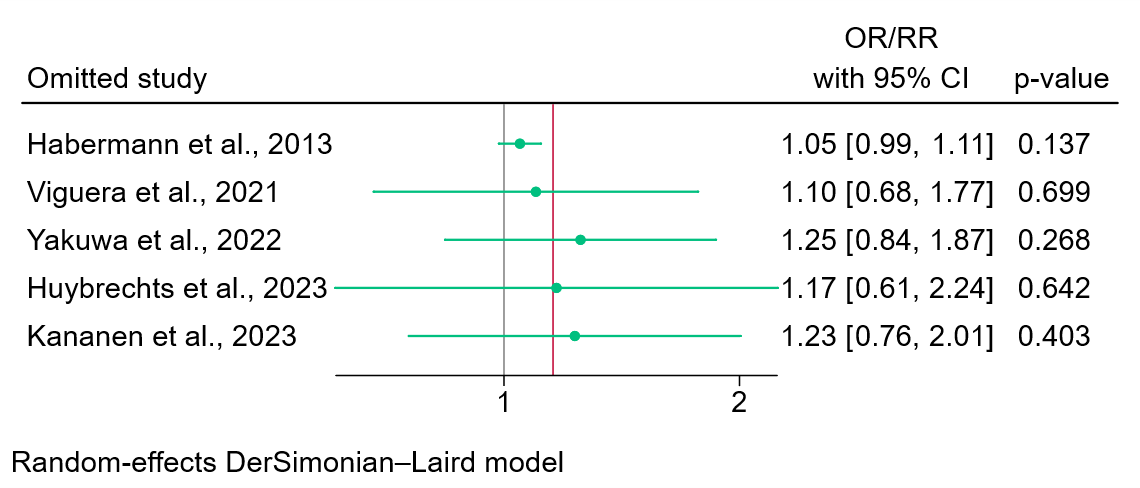


**Figure 9: Leave-one-out analysis of second-generation antipsychotics and congenital malformations**


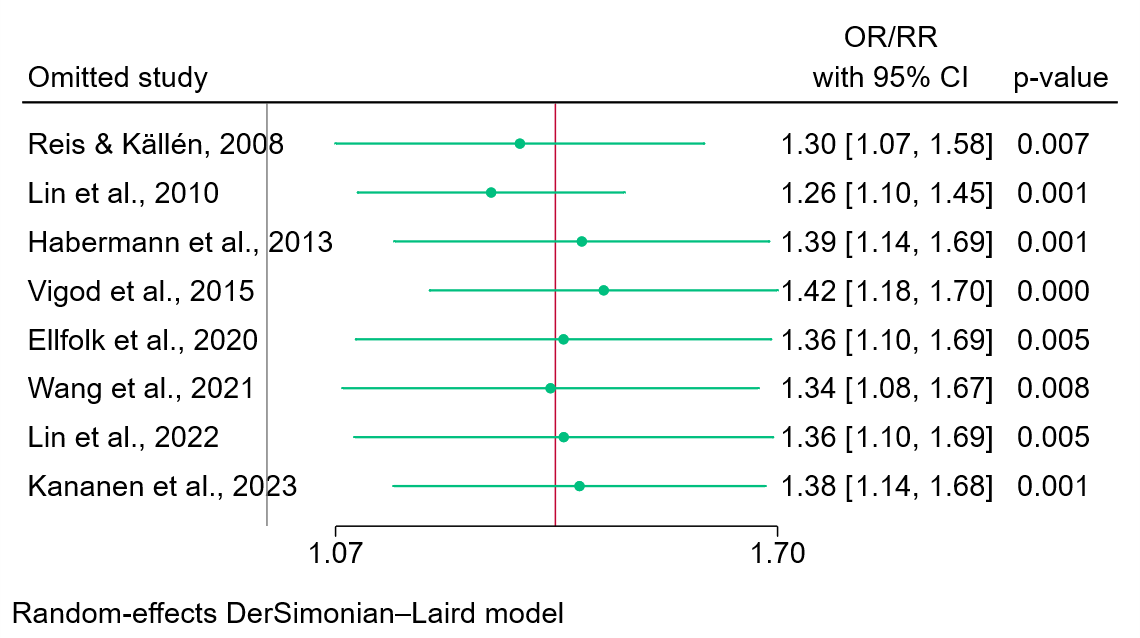


**Figure 10: Leave-one-out analysis for antipsychotic exposure and preterm birth**


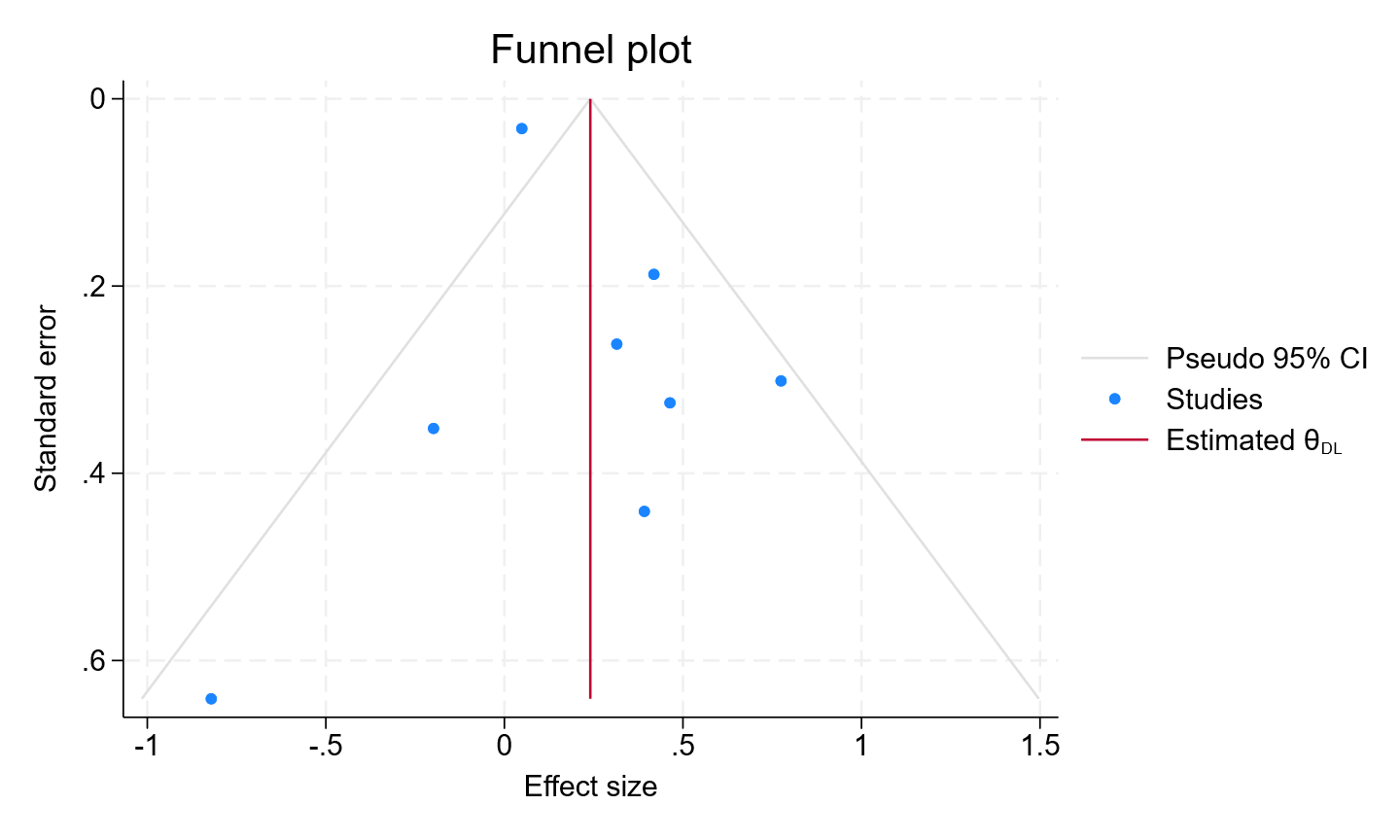


**Figure 11: Funnel plot of effect size of risk of congenital malformations following in utero antipsychotic exposure (log odds)**
